# Unusual placement of intrathecal baclofen pumps: report of two cases

**DOI:** 10.1007/s00701-015-2636-9

**Published:** 2015-11-23

**Authors:** Oliver Devine, Andrew Harborne, William B. Lo, Daniel Weinberg, Mahesh Ciras, Rupert Price

**Affiliations:** The Medical School, University College London, Gower Street, London, WC1E 6BT UK; Hull Royal Infirmary, Anlaby Rd, Kingston upon Hull, HU3 2JZ UK; Department of Neurosurgery, Queen Elizabeth Medical Centre, Birmingham, B15 2TH UK; Salford Royal NHS Foundation Trust, Stott Lane, Salford, M6 8HD UK; Department of Neurosurgery, University Hospital of North Staffordshire, Newcastle Road, Stoke-on-Trent, ST4 6QG UK; Department of Rehabilitation and Musculoskeletal Medicine, University Hospital of North Staffordshire, Newcastle Road, Stoke-on-Trent, ST4 6QG UK

**Keywords:** Baclofen, Intrathecal device, Intrathecal baclofen, Spasticity, Cerebral palsy, Spastic diplegia

## Abstract

Intrathecal baclofen delivery via implantable pump represents an important modality for symptomatic relief in patients with chronic spasticity. Pumps are routinely implanted subcutaneously in the anterior abdominal wall. We describe two unusual cases where skin-related complications necessitated revision surgery in order to relocate the pump to alternative sites. The first patient was an international power canoeist, whose strenuous exercise programme interfered with his pump’s original siting. The second patient was a cachectic university student with a history of cerebral palsy, who maintained low body mass despite attempted weight gain. The relocation of these two intrathecal devices to the medial compartment of the right thigh and right iliac fossa, respectively, is described.

## Introduction

Spasticity is regarded as a disorder of muscle tone secondary to a hypersensitivity to the stretch reflex. It leads to excessive muscle resistance to passive movement velocity, resulting in hypertonia and is a characteristic feature of upper motor neuron lesions such as cerebral palsy (CP) and hereditary spastic diplegia (HSD). In these conditions, damage to descending inhibitory neurons results in a failure to inhibit spinal motor neuron pools [1]. Whilst spasticity is a defining feature of congenital conditions such as CP and HSD, it can also be acquired later in life with a prevalence of 28–38 % in stroke, 41–66 % in multiple sclerosis and 13 % in patients following neurological trauma [2]. Managing spasticity is essential for optimising patient pain, mobility and function.

Baclofen is a γ–aminobutyric acid (GABA) analogue that binds to presynaptic GABA_B_ (G protein-coupled) receptors in the dorsal horn of the spinal cord. It inhibits neurotransmission in the spinal cord, leading to muscle relaxation. Despite high oral bioavailability, the anti-spasmodic efficacy of baclofen is limited by systemic side effects, necessitating a targeted approach. This has led to the development of intrathecal baclofen (ITB) delivery via implantable pumps which infuse baclofen continuously into the subarachnoid space. Pioneering work by Penn and Kroin [3, 4] in the 1980s resulted in successful use of ITB pumps to alleviate spasticity of spinal origin. The technique was widely adopted and achieved symptomatic relief with minimal systemic side effects [5]. ITB also had the added advantage of being reversible and programmable to a wide range of dosing regimens. A recent systematic review has confirmed a statistically significant decrease in spasticity following intrathecal baclofen pump use [6] with a typical complication rate of between zero and 2.24 complications per implant [7]. Unfortunately intrathecal therapy is costly with a recent study estimating the 5-year cost to be approximately $49,000 relative to alternative therapy [8]. In the UK, the National Commissioning Board has defined strict criteria for the funding of intrathecal pumps [9].

Typically, ITB pumps are sited subcutaneously in the anterior abdominal wall. In patients with relatively little subcutaneous adipose tissue (particularly in children [10]), ITB pumps can be sited in the subfascial plane. This technique, pioneered by Grabb and Pittman [11] in a paediatric cohort in 1998, provides greater stability for the device with lower tension placed on the overlying abdominal incision. As such, this approach has reduced instances of implant-site skin dehiscence and has a favourable aesthetic appearance—particularly in underweight patients. Despite these two widely practiced methods, a minority of patients present with unique circumstances not amenable to either placement solution. In these cases, there is limited experience of alternate placement sites described in the literature. Herein, we report on two patients in whom alternative sites for pump placement were sought, with subsequent good clinical outcomes.

## Case reports

### Case 1

A 41-year-old man with a background of hereditary spastic paraplegia was not tolerating the side effects of oral baclofen and underwent ITB pump placement subcutaneously in the right lower quadrant of the abdomen. He regularly competes as an international power canoeist, and has a muscular build.

**Fig. 1:**
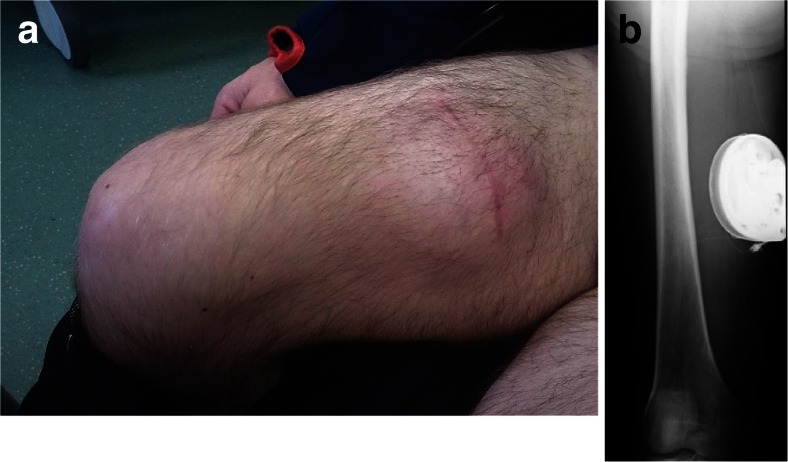
**a** Subcutaneous location of intrathecal baclofen pump device in the right medial thigh 5 months after surgery. **b** Radiograph showing the subcutaneous placement of the intrathecal baclofen pump device with associated catheter located in the right medial thigh. The catheter is seen passing superiorly from the device towards the right neck of femur

Ten months following ITB placement, he attended clinic with a history of pump site discomfort. On examination, the implanted pump had eroded a substantial amount of subcutaneous adipose tissue and the overlying skin was erythematous and tender. C-reactive protein (CRP) and erythrocyte sedimentation rate (ESR) were both within normal range. Whilst the overlying skin was intact on presentation, computed tomography demonstrated a fluid collection posterior to the pump. The fluid was aspirated with a needle, and was confirmed sterile on Gram stain microscopy and culture; this fluid was sampled for microbiological analysis and determined to be sterile. Twelve weeks later, the pump was re-sited to the right medial thigh. Intraoperatively, the pump was disconnected and removed from the original site. There was no evidence of infection in the pump cavity. A medial mid-thigh transverse incision was made and a subcutaneous pouch was created, into which the original pump was inserted. A new catheter was connected to the pump. It was first tunnelled out through an intermediate proximal thigh incision, in which the tubing was anchored to the muscle fascia (Fig. 1). Subsequently, the same catheter was tunnelled to the original abdominal pouch, where it was connected to the original distal spinal catheter with a straight connector. The pouch was irrigated with gentamicin solution and closed in layers.

Eight months later, the patient presented to his general practitioner complaining of swelling around the revised placement site. For the next 6 months he described intermittent episodes of warmth and erythema at the pump site that worsened during his physical training regime. Towards the end of this period we reviewed the patient in clinic and concluded that this swelling was likely precipitated by exercise. Ten days subsequent to this clinic visit, he was admitted due to increased swelling, pain and ulceration at the pump site. CRP, ESR and white blood cell count (WBC) were all normal. He underwent further surgery: the lateral part of the wound was reopened and thin, inflamed skin edges excised. The wound was washed and closed. Microbiological assessment of the excised skin edges revealed no evidence of infection.

At 1-month post-operative follow-up, evidence of dehiscence was still present along with underlying haematoma formation. Thinning of skin and small discharging vesicles were noted lateral to the scar. The patient underwent further surgery under the plastic surgery and neurosurgery team. The wound was debrided and washed out. A new proximal pocket was developed superficial to the deep fascia anterior to the thigh and the pump was re-sited for a second time. At post-operative follow-up, the patient was well and the wound was well healed. Six months later, there was no collection surrounding the pump, the patient was receiving regular ITB pump refills and is able to train without difficulty.

### Case 2

**Fig. 2:**
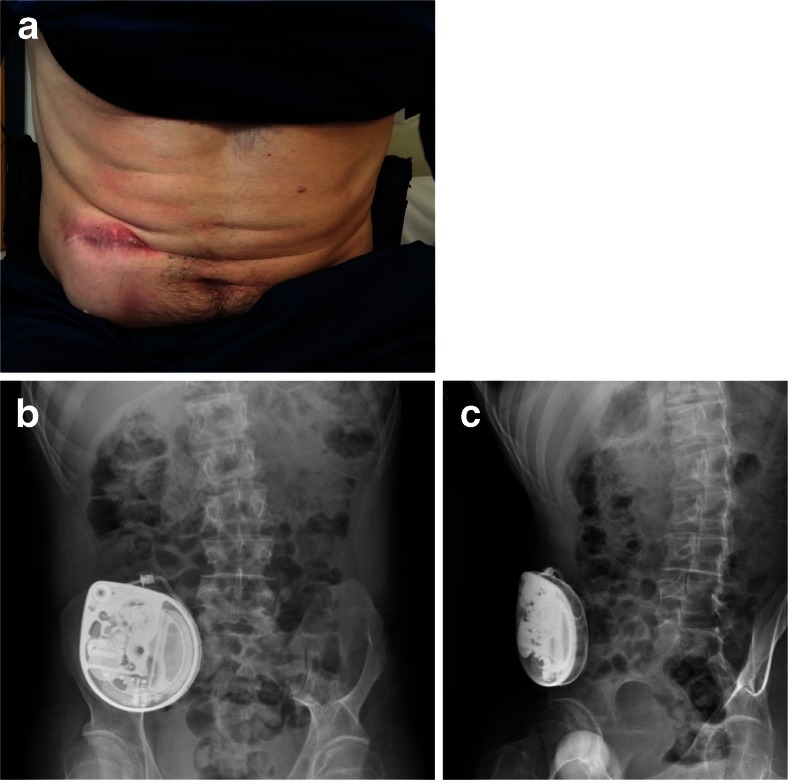
**a** Dehiscence of scar overlying the original site of intrathecal baclofen pump placement, 4 years after initial surgery. **b** Erect abdominal radiograph showing the original intrathecal baclofen pump device can be seen in the right lower quadrant of the abdomen with its catheter tracking laterally and posteriorly towards the right flank and onwards to the spinal column. **c** Lateral view abdominal radiograph depicting the paucity of subcutaneous tissue overlying the original pump site in the right lower quadrant of the abdomen

A 30-year-old man with a background of cerebral palsy and spastic diplegia had a right flank ITB pump inserted 7 years previously. During a routine follow-up clinic, the ITB pump was visibly protruding through the subcutaneous tissue of the abdominal wall (Fig. 2). This complication occurred following a period of significant weight loss after his recent move to university. Otherwise, the patient was apyrexial and there was no erythema, purulent discharge or raised temperature in the skin. WBC and CRP were normal.

**Fig. 3:**
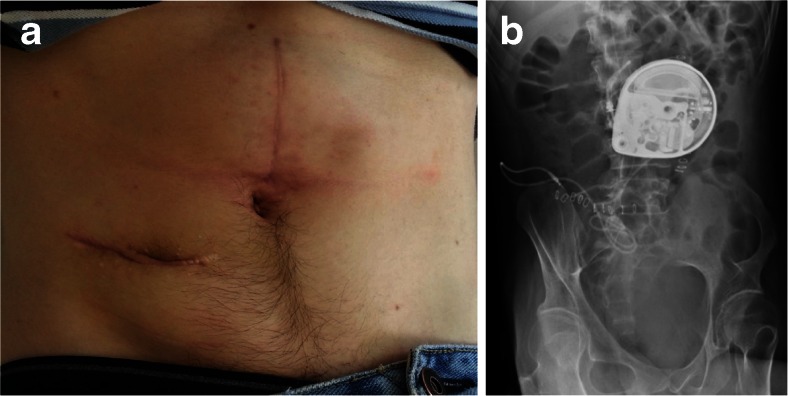
**a** Revised pump site 6 months postoperatively. Significantly less protrusion is seen with a subsequent decrease in tension on the overlying skin. **b** Erect abdominal radiograph depicting the re-sited baclofen pump in the posterior rectus fascial pouch. The catheter can be seen tracking into the right flank and onwards towards the spinal column

Six days later, the patient underwent a re-siting procedure. The old transverse wound was opened and the original pump was dissected from its capsule. It was then inserted into the potential space developed between the superior rectus abdominus and the posterior rectus sheath. The pump was anchored to the posterior rectus sheath with polyester suture. A new proximal catheter was attached to the pump and brought out superficial to the superficial linea alba, where it was connected to the old distal catheter using a straight connector (Fig. 3). The wound was closed in layers.

The patient was monitored in the ITB pump clinic, where he received regular refills. Twelve months later, the pump was stable and the wound remained intact.

## Discussion

Intrathecal baclofen delivery via an implanted pump is a safe, effective and reversible treatment for spasticity, with minimal systemic side effects. However, it requires a surgical procedure of pump and catheter insertion and regular refilling of the pump with baclofen. Therefore, it carries similar risks associated with all implanted foreign bodies. These include infection, both at the pump site and lumbar spinal region, skin irritation and wound dehiscence in addition to pump failure, catheter dysfunction and pump hypermobility.

We described two cases of an ITB pump eroding through the abdominal wall, requiring re-siting procedures. Both patients presented a surgical challenge in terms of finding an alternative pump site adequate to their body habitus and unique lifestyles. The first patient was an international power canoeist with a muscular build and low body fat. Considering his regular participation in strenuous exercise, a primary repair of the original abdominal site would be prone to further erosion. We considered the possibility of an infraclavicular placement in both patients; however, it was thought that a site overlying the pectoralis muscles would carry similar risks to the original abdominal placement. In our first patient, we replaced the pump in the unusual location of the right medial thigh (Fig. 1). Because the patient was paraplegic, this site had much less overlying skin movement and, thus, risk of erosion. In our second patient, with relatively less regular strain placed on the abdominal region, a location tethered to the rectus sheath was more appropriate and aesthetically appealing (Fig. 3).

While the abdomen remains the most common site for ITB pump insertion, as the present two cases demonstrated, occasionally alternatives need to be sought, due to various unique anatomical reasons. An ideal ITB pump implant site should be: (1) situated deep to an area of relatively ‘clean’ skin, away from creases; (2) contained within subcutaneous adipose tissue; (3) away from a pressure point or bony prominence; (4) positioned in such a way to allow uncomplicated refilling. These general rules mirror those used in the alternative placement of other medical devices such as cardiac pacemakers and implantable external defibrillators. We advise that in cases of pump site skin complication, the surgical strategy should be personalised, taking into account each patient’s anatomy and social requirement.

These two cases illustrate the circumstances that can threaten the sustainability of an existing intrathecal pump placement. These cases, and the clinical decision-making surrounding their resolution, represent a logical starting point for the holistic management of patients with similar circumstances that may preclude traditional siting of an intrathecal pump.

## References

[1] Bar-On L, Molenaers G, Aertbeliën E, Van Campenhout A, Feys H, Nuttin B, Desloovere K (2015). Spasticity and its contribution to hypertonia in cerebral palsy. Biomed Res Int.

[2] Nair KPS, Marsden J (2014). The management of spasticity in adults. BMJ.

[3] Penn RD, Kroin JS (1984). Intrathecal baclofen alleviates spinal cord spasticity. Lancet.

[4] Penn RD, Kroin JS (1987). Long-term intrathecal baclofen infusion for treatment of spasticity. J Neurosurg.

[5] McIntyre A, Mays R, Mehta S, Janzen S, Townson A, Hsieh J, Wolfe D, Teasell R (2014). Examining the effectiveness of intrathecal baclofen on spasticity in individuals with chronic spinal cord injury: a systematic review. J Spinal Cord Med.

[6] Butler C, Campbell S, Adams R (2000). Evidence of the effects of intrathecal baclofen for spastic and dystonic cerebral palsy. Dev Med Child Neurol.

[7] Stetkarova I, Yablon SA, Kofler M, Stokic DS (2010). Procedure- and device-related complications of intrathecal baclofen administration for management of adult muscle hypertonia: a review. Neurorehabil Neural Repair.

[8] De Lissovoy G, Matza LS, Green H, Werner M, Edgar T (2007). Cost-effectiveness of intrathecal baclofen therapy for the treatment of severe spasticity associated with cerebral palsy. J Child Neurol.

[9] National Institute for Health and Clinical Excellence (2012) Spasticity in children and young people with nonprogressive brain disorders http://www.ncbi.nlm.nih.gov/books/NBK116583/. Accessed 26 Oct 2015

[10] Ammar A, Ughratdar I, Sivakumar G, Vloeberghs MH (2012). Intrathecal baclofen therapy—how we do it. J Neurosurg Pediatr.

[11] Grabb P, Pittman A (1998) Subfascial placement of baclofen pumps. Presented at the Annual Meeting of the Joint Section of Pediatric Neurological Surgery, Indianapolis

